# Artificial Intelligence in Bacterial Infections Control: A Scoping Review

**DOI:** 10.3390/antibiotics14030256

**Published:** 2025-03-02

**Authors:** Rasha Abu-El-Ruz, Mohannad Natheef AbuHaweeleh, Ahmad Hamdan, Humam Emad Rajha, Jood Mudar Sarah, Kaoutar Barakat, Susu M. Zughaier

**Affiliations:** 1 College of Health Sciences, QU Health, Qatar University, Doha P.O. Box 2713, Qatar; rasha@qu.edu.qa; 2 College of Medicine, QU Health, Qatar University, Doha P.O. Box 2713, Qatar; ma1908120@qu.edu.qa (M.N.A.); ah1904442@qu.edu.qa (A.H.); hr2003606@qu.edu.qa (H.E.R.); 3 College of Medicine, University of Jordan, Amman P.O. Box 11942, Jordan; jod0226581@ju.edu.jo; 4 College of Pharmacy, QU Health, Qatar University, Doha P.O. Box 2713, Qatar; kb1905219@qu.edu.qa

**Keywords:** infectious diseases, artificial intelligence, communicable diseases, bacterial infection control, infection and prevention

## Abstract

**Background/Objectives**: Artificial intelligence has made significant strides in healthcare, contributing to diagnosing, treating, monitoring, preventing, and testing various diseases. Despite its broad adoption, clinical consensus on AI’s role in infection control remains uncertain. This scoping review aims to understand the characteristics of AI applications in bacterial infection control. **Results**: This review examines the characteristics of AI applications in bacterial infection control, analyzing 54 eligible studies across 5 thematic scopes. The search from 3 databases yielded a total of 1165 articles, only 54 articles met the eligibility criteria and were extracted and analyzed. Five thematic scopes were synthesized from the extracted data; countries, aim, type of AI, advantages, and limitations of AI applications in bacterial infection prevention and control. The majority of articles were reported from high-income countries, mainly by the USA. The most common aims are pathogen identification and infection risk assessment. The most common AI used in infection control is machine learning. The commonest reported advantage is predictive modeling and risk assessment, and the commonest disadvantage is generalizability of the models. **Methods**: This scoping review was developed according to Arksey and O’Malley frameworks. A comprehensive search across PubMed, Embase, and Web of Science was conducted using broad search terms, with no restrictions. Publications focusing on AI in infection control and prevention were included. Citations were managed via EndNote, with initial title and abstract screening by two authors. Data underwent comprehensive narrative mapping and categorization, followed by the construction of thematic scopes. **Conclusions:** Artificial intelligence applications in infection control need to be strengthened for low-income countries. More efforts should be dedicated to investing in models that have proven their effectiveness in infection control, to maximize their utilization and tackle challenges.

## 1. Introduction

Since the nineteenth century, infection prevention and control (IPC) has become an important concept to tackle antimicrobial resistance (AMR) and healthcare-associated infections (HAIs) [1]. The ongoing advancement of IPC is critical to prevent disease transmission and outbreaks [2]. An evidence-based IPC approach has been undertaken in the recent decade to safeguard patients and health workers from preventable infections [2]. The European Centre for Disease Prevention and Control (ECDC) and the World Health Organization (WHO) declared in Geneva 2017 the core components for evidence-based strategies that target IPC and reduce HAIs [3]. The included national core components are to develop active national programs that tackle infections, strengthen the reliance of evidence-based guidelines, impose the education and training of healthcare workers, enhance mechanisms of timely data management and feedback, and clinically evaluate the hygiene monitoring programs [3]. The concept of IPC is not only entailed to HAI but also applies on community-associated infections (CAIs), which is another aspect of global public health concern that focuses on community outbreaks and the spread of antimicrobial-resistant organisms across communities [4]. Early detection, effective treatment, and surveillance are the three key aspects in IPC to reduce health complications and mortality resulting from HAIs and CAIs [5,6,7,8,9,10,11,12]. Although not well-understood, new technologies and artificial intelligence (AI) play vital roles in enhancing healthcare surveillance maneuvers and positively impacting patients’ health [13]. AI possess the ability to facilitate decision-making, hospital management, and early outbreak detection for prevention carried out by physicians, administrators, and public health officials [14,15]. Some studies reported that the utilization of AI in IPC optimizes resources, enhance sensitivity of diseases detection, and aids the practices of personalized medicine [16,17]. AI relies on data that are entered into computational models to enhance the sought health outcome [18]. Common AI modalities used in healthcare are Machine Learning (ML) and Deep Learning (DL). These algorithms and models were used in some research to predict HAIs through risk factors and to improve patients’ health outcomes [5,19,20]. This scoping review aims to understand the characteristics of AI applications in bacterial infection control. The review focuses on AI applications that contribute to infection prevention and control practices studied in both community-associated and healthcare-associated bacterial infections.

## 2. Results

### 2.1. Search Results

**Figure 1:**
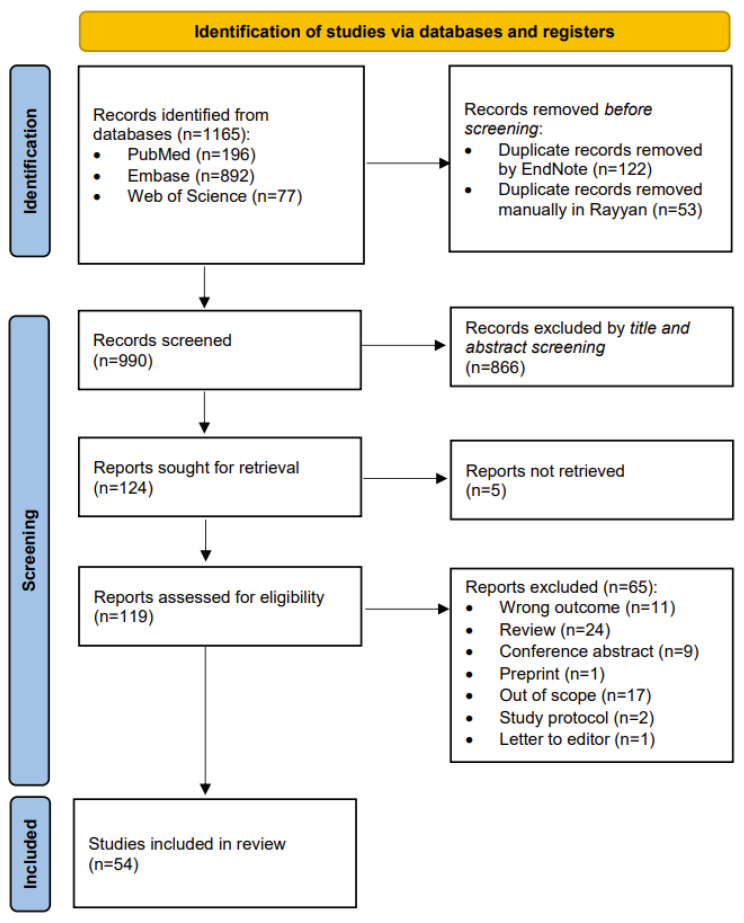
PRISMA-ScR chart.

The search yielded a total of (n = 1165) articles from PubMed (n = 196), Embase (n = 892), and Web of Science (n = 77) (Figure 1). The duplicates were identified and removed by Endnote v20.2.1 and rayyan platform (n = 127). The unique remaining citations (n = 990) were then subjected to title and abstract screening. Irrelevant citations were excluded (n = 866) as they did not cover the concept of AI in bacterial infection control. The remaining articles were selected for full-text screening (n = 124). A total of five citations were excluded as the full-text record was not available for retrieval. The remaining citations (n = 65) were excluded due to the following reasons: wrong outcome, review article, conference abstract, preprint, out of scope, study protocol, or letter to editor. The resulting articles (n = 54) were extracted and analyzed (Table 1, and Supplementary: S6).

### 2.2. Qualitative Synthesis

Qualitative synthesis was performed to understand the applications of AI in bacterial infection control. The extracted data from the citations were synthesized to construct five thematic scopes illustrated in a matrix (Table 2): countries, aim, type of AI, advantages, and limitations. The three scopes; aim, advantages, and limitations, involved coding keywords and the generation of categories that helped in mapping and summarizing the extracted articles.

#### 2.2.1. Scope of Countries

The countries were labeled according to the World Bank’s income classifications for 2023 [74]. In our comprehensive review of 54 articles, 37 studies (68.5%) originated from high-income countries [20,21,22,23,25,26,27,29,30,31,32,33,34,37,38,39,40,41,42,43,45,49,51,52,53,54,57,58,59,60,64,65,66,68,69,73,75]. Upper-middle-income countries contributed to nine studies (16.7%) [28,36,46,47,56,67,70,71,72]. Conversely, lower-middle-income countries were represented by only two studies (3.7%) [24,63].

Among the remaining studies, three (5.6%) were collaborations between high-income and upper-middle-income countries [36,44,62], and one (1.8%) involved high-income and lower-middle-income countries [28]. Additionally, two studies (3.7%) were conducted on multiple countries of all levels of incomes [50,55].

The United States has emerged as the foremost contributor to this research area, publishing 16 articles on AI applications in infection control [22,23,25,26,37,40,45,49,51,54,57,58,59,60,61,64]. The majority of the studies focused on multidrug-resistant bacteria, *M. tuberculosis*, *C. difficile*, and septicemia.

China follows with eight articles [35,46,47,48,67,70,71,72] that focus on *K. pneumonia*, *Acinetobacter*, and *H. pylori*. France has also contributed with four studies [20,26,28,41]; two of the studies were collaborative with other countries and focus on *M. tuberculosis*.

Only two studies were conducted in low–middle-income countries. One study conducted in India focused on phage therapy of multidrug-resistant bacteria [24] and the other study in Bangladesh focused on community-acquired infections and household water as source of typhoid risk [63].

#### 2.2.2. Scope of Aim

**Figure 2:**
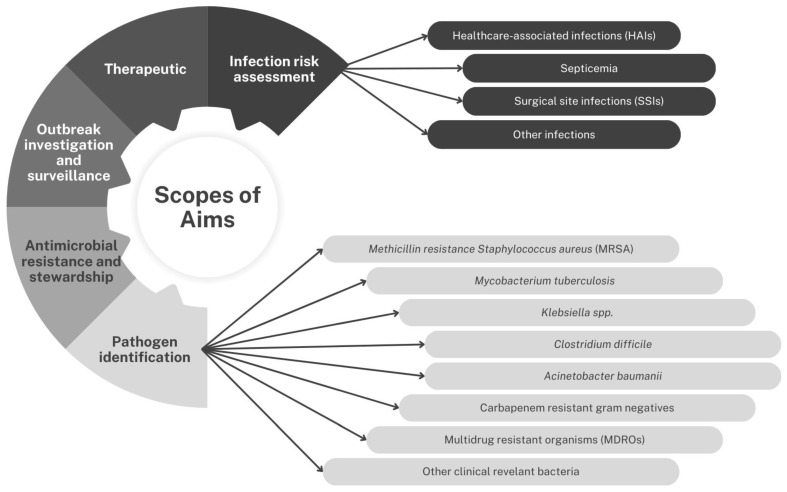
Categories of aims of AI in infection prevention and control.

The scope of the aim was constructed based on categorizing shared characteristics of the extracted articles. Five categories were generated based on coded keywords to include pathogen identification, infection risk assessment, therapeutic, outbreak investigation, and antimicrobial resistance stewardship (Supplementary: S3). However, the categories of aim are interchangeable and reflect on each other due to the relatedness of the microorganisms’ classifications (Figure 2).

##### Pathogen Identification

A total of 28 (52%) studies focused on bacterial pathogen identification, 17 of the 28 (61%) employed Machine Learning, 4 (14%) Deep Learning, 6 (21%) the hybrid approach, and 1 (4%) supervised neural network.

Methicillin Resistance *Staphylococcus aureus* (MRSA)

Eight studies focused on bacterial pathogen identification concerning MRSA [21,22,30,40,41,54,65,66]. Jeon 2022 [21] utilized AMRQuest to distinguish MRSA. Camoez 2016 [30] utilized CLINPROTOOLS and MALDI BIOTYPER to differentiate MRSA into four major clonal complexes. Hsu 2008 [40] developed an Artificial Neural Network model to predict MRSA carriage and found higher accuracy for a hospital in the USA compared to a hospital in Taiwan. The model maintained accuracy with a reduced number of risk factors, and cross-validation showed potential applicability across different healthcare settings. Jacques 2020 [41] utilized a Multi-Objective Classification Algorithm for Imbalanced Data (MOCA-I) to identify hospitalized patients at risk of testing positive for MRSA. The algorithm could correctly identify 58% of the patients who were carriers or infected with MRSA. The precision of the algorithm for MRSA was 88%. Rhodes 2023 [54] developed a Machine Learning model to predict MRSA in hospitalized patients with community-acquired pneumonia (CAP). The key predictors included ICU admission, mechanical ventilation, and recent antibiotic use. Wang 2016 [66] demonstrated that a Machine Learning-based identification system using MALDI-TOF MS analysis (ClinProTools version 3.0, Bruker Daltonik GmbH, Bremen, Germany) can accurately classify major multilocus sequence types of MRSA. Wang 2018 [65] developed a Machine Learning-based identification system for MRSA strain typing using MALDI-TOF MS spectra. The system utilized Decision Tree (DT), Support Vector Machine (SVM), and k-nearest neighbor (KNN) algorithms. Çaǧlayan Ç 2022 [22] developed a data-driven framework to identify patients likely to be colonized with Vancomycin-Resistant *Enterococci* (VRE), Carbapenem-resistant *Enterobacterales* (CRE), or MRSA upon Intensive Care Unit (ICU) admission, leveraging Electronic Health Record (EHR) data to generate timely and accurate predictions.

*Mycobacteria tuberculosis*

Four studies focused on *Mycobacteria tuberculosis*. Althomsons 2022 [25] utilized Machine Learning techniques to analyze national tuberculosis surveillance data and predict the growth of TB clusters. The model demonstrated moderate predictive accuracy. Aminian 2014 [26] developed a Knowledge-Based Bayesian Network (KBBN) for classifying *Mycobacterium tuberculosis complex* (MTBC) clades. The KBBN model demonstrated high classification accuracy. Azé 2015 [28] presented a novel highly accurate and rapid approach (TBminer) to classifying *Mycobacterium tuberculosis* complex (MTC) strains using Machine Learning and genomic data. Singh 2024, [60] identified a profile of six proteins that can accurately differentiate between healthy individuals and those with latent TB (LTBI) using Machine Learning models, particularly the Random Forest classifier.

*Klebsiella* spp.

Three studies focused on *Klebsiella* spp. Feng 2023 [35] developed and validated a Machine Learning-based prediction model for invasive *Klebsiella pneumoniae* liver abscess syndrome (IKPLAS) in patients with diabetes mellitus (DM). The model identified hemoglobin, platelet count, D-dimer level, and SOFA score as key predictors. Lapp 2021 [45] utilized Machine Learning models to evaluate the predictive power of patient characteristics and *Carbapenem-resistant Klebsiella pneumoniae* (CRKP) genomic features. The study reported that both sets of factors were similarly predictive of infection; the patient predictors included indwelling devices and kidney disease, while genomic predictors included specific genetic elements and disruptions. Lyu 2023 [48] developed a Deep Learning-based identification system that utilizes surface-enhanced Raman scattering spectroscopy to rapidly and accurately predict multidrug-resistant (MDR) *Klebsiella pneumoniae*.

*Clostridium difficile*

Three studies focused on *Clostridium difficile*. Ötleş 2023 [23] found that the Machine Learning model for identifying hospital-onset *Clostridioides difficile* infection (HO-CDI) demonstrated high accuracy (97.5%) and specificity (99.4%) when combined with swab surveillance; however, it had lower sensitivity (43.6%) and generated a significant number of false positives. Marra 2020 [49] found that the usage of Machine Learning models to predict CDI in hospitalized patients using clinical data was achievable, but the effectiveness of these models was limited, with the top models reaching an AUC of 0.6. Key predictors of CDI included age and recent antibiotic use. Panchavati 2022 [51] found that Machine Learning algorithms, particularly XGBoost, can effectively predict CDI using data from the first six hours of hospitalization, achieving high discrimination with an AUC greater than 0.8. They showed that key predictive features included age, antibiotic and proton pump inhibitor treatments, and various clinical measurements. While XGBoost outperformed Neural Networks in terms of AUROC and specificity, the Neural Networks achieved higher sensitivities.

*Acinetobacter baumannii*

Two studies focused on *Acinetobacter baumannii.* Doan 2015 [34] utilized hidden Markov models (HMMs) to investigate the transmission dynamics of *Acinetobacter baumannii* in ICUs across three hospitals. Zeng 2024 [71] developed a Machine Learning classification model that effectively distinguishes between pulmonary infection and colonization of *Acinetobacter baumannii* by analyzing time-series chest radiographs and laboratory data. The model incorporated data from multiple time points and demonstrated superior performance with an AUC of 0.850.

Multidrug-Resistant Organisms (MDROs)

Four studies focused on MDROs. Çaǧlayan 2022 [22] developed a Machine Learning-based predictive framework that accurately identified patients likely to be colonized with multidrug-resistant organisms (MDROs) upon ICU admission, achieving high sensitivity and specificity. The MDROs identified in this study were MRSA, Carbapenem-Resistant *Enterobacteriaceae* (CRE), and VRE. Key predictors for MDRO colonization included long-term care facility exposure and current diagnoses of skin or any contraction of active infectious disease either viral, parasitic, or bacterial in origin. The framework addresses class imbalance in clinical datasets and enables real-time alerts for timely infection control measures, thereby enhancing patient safety and optimizing resource use in critical care settings. Jacques 2020 [41] utilized MOCA-I, which demonstrated a recall of 62% for MDR bacteria with a risk score cut-off above 0.85. The precision for MDR bacteria was 69%. Wang 2023 [67] developed a Machine Learning-based identification system using a Backpropagation Neural Network (BPNN) to predict MDRO infection in critically ill patients. The model was trained and validated on data from the ICU, identifying nine significant risk factors for MDRO infection, which include duration of hospitalization and ICU stay, long-term bed rest, prior antibiotic use, APACHE II score, history of invasive operations, quantity of antibiotics administered, chronic lung disease, and hypoproteinemia. The BPNN model demonstrated high predictive accuracy with AUC values above 0.8. Gouareb 2023 [76] demonstrated the application of Graph Neural Networks (GNNs) in predicting patients at risk of colonization by multidrug-resistant *Enterobacteriaceae*, achieving high AUC scores and outperforming traditional Machine Learning baselines. The model’s performance was consistent across different species, specimen types, lengths of stay, and resistance profiles.

Carbapenem-resistant Gram-negative organisms

Two studies focused on carbapenem-resistant Gram-negative organisms. Liang 2024 [46] developed Machine Learning models to predict carbapenem-resistant Gram-negative bacterial bloodstream (CR-GNB) infections in ICU patients. The Random Forest model showed the highest predictive accuracy with an AUC of more than 0.85. The identified risk factors included mechanical ventilation, invasive catheterization, and carbapenem use history. Liang 2022 [47] developed a Machine Learning-based prediction model to identify patients at risk of CR-GNB carriage in an ICU. The model included 16 significant predictive variables.

Three studies focused mainly on *Enterobacteriacae* carbapenem resistance. Goodman 2019 [37] investigated the prevalence of colonization with Carbapenem-Resistant *Enterobacteriaceae* (CRE) and other Carbapenem-Resistant Organisms (CROs), including Carbapenemase-Producing Organisms (CPOs), at the time of admission to hospital units. The models were successful in identifying patients with a history of CRO-positive cultures and those using proton pump inhibitors as having a higher risk of colonization. Çaǧlayan 2022 [22] developed an XGBoost algorithm for predicting CRE colonization, which achieved a sensitivity of 73% and a specificity of 77%, indicating its effectiveness in identifying patients colonized with CRE while maintaining a reasonable rate of correct negative predictions. Freire 2022 [36] developed a Machine Learning-based predictive model to identify liver transplant patients at high risk for CRE colonization. The model identified key risk factors such as recent antibiotic use and hepato-renal syndrome. With a sensitivity of 66% and a specificity of 83%, the model showed promise in predicting CRE colonization, particularly with the Random Forest classifier algorithm outperforming others.

Other clinically relevant bacteria

Three studies focused on other clinically significant bacteria. Hattori 2020 [39] developed a Machine Learning-based system capable of rapidly identifying single cells of five clinically important pathogenic bacteria, *Staphylococcus aureus*, *Pseudomonas fluorescens*, *Salmonella enterica*, *Escherichia coli*, and *Bacillus cereus*, using a low aspect ratio pore sensor and resistive pulse analysis. This innovative approach leveraged the unique motility of bacteria, enabling the system to classify them with an impressive 91% accuracy within milliseconds. Zhong 2022 [72] found that the YOLO v5 Machine Learning model could accurately detect the *Helicobacter pylori* coccoid form (HPCF), achieving performance levels comparable to or surpassing those of experienced pathologists. Yan 2022 [70] developed an Integrated Promoter Markov Discriminant (IPMD) algorithm that effectively predicted *Escherichia coli* promoter sequences. The IPMD algorithm outperformed other methods by integrating multiple features and achieving a high overall accuracy of 89.2%. The model’s stability across different training and test set ratios demonstrates robustness, suggesting the potential for generalization to other promoter prediction tasks.

##### Infection Risk Assessment

Thirteen studies focused on infection risk assessment, all studies were conducted in clinical sites. De Bruin 2017 [33], developed a rule-based system using Knowledge Discovery and Semantic Analysis to process medical knowledge for analyzing microbiological laboratory test results. Their ultimate goal was to facilitate electronic surveillance of healthcare-associated infections (HAIs). Other studies used machine learning; Jakobsen 2024 [42] utilized Bayesian Network models to stratify risk for hospital-acquired urinary tract infections (HA-UTI) within the first 24 hours of patient admission. Rabhi 2018 [20] developed a model that incorporate word embeddings (word2vec) to better detect healthcare-associated infections (HAIs). Ratzinger 2015 [52] employed tools such as Weka, R, and MDCalc bvba to effectively differentiate between Gram-positive and Gram-negative bacteremia. Rennert-May 2022 [53] used Python and Scikit-Learn modeling to identify complex surgical site infections (SSIs) following cardiac implantable electronic device (CIED) implantation. Savin 2018 [56] applied tree-based machine learning algorithms, leveraging XGBoost to determine the incidence of healthcare-associated ventriculitis and meningitis (HAVM) in a neuro-ICU setting. Schinkel 2022 [57] also employed XGBoost to predict blood culture outcomes in the emergency department. Seheult 2023 [58] developed machine learning decision tree algorithm (PittUDT) using R “rpart” package to optimize urinalysis parameters for predicting urine culture positivity. Shohat 2020 [59] developed Random Forest to predict outcomes following irrigation and debridement (I&D) surgery for prosthetic joint infection. Tadesse 2023 [63] used decision tree modeling, “rpart.plot” that examines the association between household water, sanitation, and hygiene (WASH) conditions and typhoid risk in urban slums. Tsurumi 2023 [64] applied the Least Absolute Shrinkage and Selection Operator (LASSO) algorithm to develop a predictive tool for bloodstream infections in children with burns. Waterhouse 2011 [68] employed Bayesian Network analysis to assess the risk of MRSA transmission in relation to hospital overcrowding. Wu 2023 [69] developed automated approach based on XGBoost to detect complex surgical site infections (SSIs) following total hip and knee arthroplasty.

Healthcare-associated infections (HAIs)

Four studies focused on healthcare-associated infections. de Bruin 2017 [33] developed a library to analyze microbiological laboratory test results, aiming to enhance electronic surveillance of healthcare-associated infections (HAIs). Rabhi 2019 [20] found that CNNs significantly outperformed conventional ML models in detecting HAIs, achieving an F1 score of 97.7% and an AUC of 99.8%. The CNN model demonstrated a good balance between sensitivity (0.962) and specificity (0.937) and proved robust across different hospital datasets. Jakobsen 2024 [42] developed Bayesian Network (BN) models to predict hospital-acquired urinary tract infection (HA-UTI) risk within 24 hours of admission. The model achieved the highest performance with an AUC of 0.746. Savin 2018 [56] found that tree-based Machine Learning algorithms, specifically Random Forest and XGBoost, helped in identifying risk factors for healthcare-associated ventriculitis and meningitis (HAVM) in high-risk neuro-ICU patients. The key risk factors identified were external ventricular drains, craniotomy, superficial surgical site infections (SSIs), and cerebrospinal fluid leakage.

Septicemia

Three studies focused on septicemia and bacteria. Tsurumi 2023 [64] presented a Machine Learning-based approach for predicting bloodstream infections in children with severe burns, using a multi-biomarker panel model developed through the Least Absolute Shrinkage and Selection Operator (LASSO) algorithm. This model achieved high predictive accuracy, outperforming traditional clinical predictors, and showed even greater accuracy when combined with clinical data. Ratzinger 2015 [52] found that the K-Star and Random Forest algorithms demonstrated limited predictive power (AUC of 0.675) and poor calibration in distinguishing between Gram-positive and Gram-negative bacteremia. The study concluded that routine laboratory parameters, even when analyzed with advanced Machine Learning techniques, are not reliable predictors for the type of bacteremia and should not be used for clinical decision-making. However, the study does highlight the potential of these models for infection risk assessment, indicating a need for further research to improve predictive accuracy and clinical applicability. Schinkel 2022 [57] developed and validated a Machine Learning model, specifically using XGBoost, to predict blood culture outcomes and assess infection risk in emergency departments. The model achieved high performance metrics, with an AUC of 0.81 in the test set and similar results in external validations across different hospitals. Key predictors included temperature, creatinine, and C-reactive protein. The model demonstrated the potential to safely withhold blood culture analyses in at least 30% of patients, reducing unnecessary tests and associated costs. A real-time prospective evaluation confirmed its practical applicability, suggesting that the model could significantly improve diagnostic efficiency, infection risk assessment, and resource utilization in clinical settings.

Surgical site infection (SSI)

Two studies focused on surgical site infection. Rennert-May 2022 [53] found that Machine Learning models significantly outperformed traditional methods in identifying complex SSIs following cardiac implantable electronic device implantation. The Machine Learning model achieved an AUC of 96.8%. The model demonstrated excellent sensitivity and specificity, effectively tracking infection trends over time. Wu 2023 [69] not only focused on the development of machine learning models for the detection of SSIs but also highlighted the potential for these models to be used in risk assessment. The high performance of the XGBoost models, as indicated by an AUC of 0.906 and F1 score of 0.79, suggests their potential utility in risk stratification and early intervention.

Other infections

Three studies focused on other infections. Seheult 2023 [77] developed and validated the PittUDT Machine Learning Decision Tree algorithm to optimize urinalysis parameters for predicting urine culture positivity and assessing infection risk. The algorithm demonstrated high predictive accuracy with a negative predictive value above 90%, effectively reducing unnecessary urine cultures and supporting cost savings and antimicrobial stewardship; the algorithm showed robust performance and broad applicability. Shohat 2020 [59] developed and validated a Machine Learning-based tool to predict the outcome of irrigation and debridement surgery for prosthetic joint infection. The model identified key predictors of treatment failure, including higher serum CRP levels, positive blood cultures, and MRSA infection. The algorithm demonstrated good accuracy (AUC = 0.74) and was validated through cross-validation. This tool offered practical means to enhance personalized treatment. Tadesse 2023 [63] developed a Machine Learning-based composite to classify households in urban slums into “Better” and “Not Better” categories based on private toilet facilities, safe drinking water, and water filters. The findings revealed that living in “Better” WASH households was associated with a 38% reduction in typhoid risk. The model demonstrated good predictive performance and was validated in a separate subpopulation.

Therapeutic Options

Six studies focused on therapeutic options. Aggarwal 2023 [24] developed the PhageTB tool, which utilized Machine Learning to predict phage–host interactions through three modules: host prediction for phages, interaction assessment, and phage identification for specific bacterial hosts. The tool demonstrated superior performance compared to existing methods, achieving high predictive accuracies across various taxonomic levels. Bournez 2023 [29] presented the development and evaluation of novel Machine Learning models CalcAMP and CalcAFP for predicting antimicrobial and antifungal activities of peptides, with a focus on short peptides under 35 amino acids; the models’ accuracies were between 75 and 88%. Cherkasov 2009 [32] utilized ANN to create predictive models for identifying small peptide antibiotics effective against MDR superbugs. Sambarey 2024 [55] developed a multimodal Machine Learning model that combines clinical, genomic, imaging, and drug resistance data to predict TB treatment outcomes with high accuracy (83%) and AUC (0.84). The model successfully identified effective drug regimens for MDR non-XDR TB, such as Bedaquiline, Clofazimine, Cycloserine, Levofloxacin, and Linezolid, and emphasized the value of synergistic drug combinations for improved results. Tacconelli 2020 [62] utilized the Random Forest algorithm to assess the risk of ESBL-GNB colonization associated with different antibiotic regimens, revealing that monotherapy with cephalosporins posed the highest risk, while combination therapies generally resulted in lower colonization rates. The analysis highlighted the need for personalized risk assessments based on individual patient histories and specific antibiotic usage patterns. Zwerwer 2024 [73] demonstrated that Machine Learning models, particularly Long Short-Term Memory (LSTM) Neural Networks, can effectively predict the need for infection-related consultations in ICU patients, with the ability to provide predictions up to eight hours in advance. The models achieved high predictive accuracy, with an AUC of (0.92).

Outbreak investigation and surveillance

Three studies focused on outbreak investigation and surveillance. Atkinson 2023 [27] applied Machine Learning and graph theory to enhance the investigation of a nosocomial outbreak of Vancomycin-Resistant *Enterococci* (VRE). It identified key risk factors for VRE colonization, including age, ICU admission, comorbidity score, antibiotic exposure, and patient room transfers. Utilizing a Decision Tree approach, these findings were validated, and network graph analysis uncovered three primary transmission pathways: healthcare personnel, medical devices, and patient rooms. Cheah 2018 [31] employed a mathematical model to analyze the impact of active surveillance and contact isolation on VRE transmission in a hematology–oncology ward. The findings suggest that most VRE acquisitions were attributed to background rates rather than patient-to-patient transmission, with an estimated 31% of cases due to cross-transmission, albeit with significant uncertainty. Sundermann 2021 [61] found that combining whole genome sequencing (WGS) surveillance with ML algorithm effectively identified a previously undetected outbreak of *Pseudomonas aeruginosa* infections linked to a contaminated gastroscope. The ML algorithm accurately pinpointed gastroscopy as the transmission route.

Antimicrobial resistance and stewardship

Three studies focused on antimicrobial resistance and stewardship. Khaledi 2016 [43] utilized a Support Vector Machine to analyze transcriptome data from *Pseudomonas aeruginosa* isolates and successfully identified genetic markers that discriminate between resistant and susceptible strains, with a high classification accuracy for ciprofloxacin resistance. This approach suggest a potential tool for rapid and accurate diagnostics that could inform targeted treatment strategies against antimicrobial resistance. Khaledi 2020 [44] utilized Machine Learning to predict antimicrobial resistance in *Pseudomonas aeruginosa* using genomic and transcriptomic data. Through analyzing 414 clinical isolates, they focused on resistance to certain antibiotics including ceftazidime, meropenem, ciprofloxacin, and tobramycin. They revealed that integrating gene presence or absence and gene expression data significantly enhanced the accuracy of resistance predictions for all antibiotics except ciprofloxacin. The classifiers demonstrated high sensitivity and predictive values, with the strongest performance observed for tobramycin and meropenem. Noman 2023 [50] utilized BioWeka and Random Forest (RF) models, in which it demonstrated high accuracy in predicting AMR in *Pseudomonas aeruginosa* using whole genome sequence (WGS) data. BioWeka achieved a mean classification accuracy (≥98%), while RF achieved (≥96%) across twelve antibiotics.

#### 2.2.3. Scope of AI Type

In this scoping review exploring the application of artificial intelligence (AI) in infection control, a total of 54 studies were analyzed, employing various types of AI models and algorithms across different settings. The majority of these studies utilized Machine Learning (ML) algorithms [21,22,23,27,28,29,30,31,34,35,36,37,39,41,42,43,44,45,46,47,49,50,52,53,54,56,57,59,60,61,62,63,64,68,69,71,77], such as Logistic Regression (LR), Random Forest (RF), Support Vector Machines (SVM), Decision Trees (DTs), and eXtreme Gradient Boosting (XGBoost). Eight studies [25,32,40,51,55,65,66,73] employed hybrid approaches combining Machine Learning and Deep Learning (DL) techniques, which included models like SVM, Neural Networks, and ensemble methods. Deep Learning (DL) models alone were used in four studies [48,67,72,76], including algorithms like Convolutional Neural Networks (CNNs) and Graph Neural Networks (GNNs), showcasing their ability to learn complex patterns for infection prediction and pathogen identification. Additionally, computational biology and alignment-based methods were used in three studies [24,26,70], such as BLASTPhage, which facilitated research into therapeutic approaches like phage therapy. One study [33] incorporated knowledge discovery and semantic analysis (KD&SA), focusing on rule-based systems for processing medical knowledge. One study [20] uniquely combined Machine Learning, Deep Learning, and Natural Language Processing (NLP) to analyze vast amounts of unstructured textual data, such as patient records and medical reports.

##### Machine Learning (ML)

Machine Learning (ML) was the most frequently applied AI technology, appearing in 37 studies [21,22,23,27,28,29,30,31,34,35,36,37,39,41,42,43,44,45,46,47,49,50,52,53,54,56,57,59,60,61,62,63,64,68,69,71,77]. These algorithms provided robust predictions, diagnostic capabilities, and decision support for infection control. The following ML algorithms were used:

AMRQuest software, v.2.1 was employed in one study [21]. AMRQuest focused on pathogen identification, especially detecting antibiotic-resistant organisms such as MRSA. This software’s role in the studies was crucial for the rapid and accurate identification of resistant pathogens, which is essential for timely intervention and preventing the spread of infections in clinical settings.

Logistic Regression (LR) was featured in 12 studies [22,23,24,25,35,45,47,49,53,71,73], and it was employed for predictive modeling of infection risk, assessing patient outcomes, and identifying potential healthcare-associated infections (HAIs). This algorithm’s simplicity and interpretability made it valuable in forecasting patient risk and guiding early interventions.

Random Forest (RF) was used in 13 studies [22,24,25,35,36,45,46,47,49,56,59,62,73], and it was applied to predict high-risk infection cases by analyzing multiple variables simultaneously. Its ability to handle imbalanced datasets made it effective in identifying HAIs, especially in clinical scenarios with complex patient data.

Support Vector Machines (SVMs) were used in 11 studies [22,24,25,35,43,44,45,46,49,65,70] primarily for classification tasks, helping improve diagnostic accuracy by distinguishing between infection and non-infection cases. The algorithm’s capacity to handle high-dimensional data enabled better discrimination of complex patient profiles.

Decision Trees (DTs) were applied in eight studies [24,25,27,35,37,63,65,77] as these were important for stratifying patients into different risk categories based on their likelihood of acquiring infections. Its transparent decision-making process allowed healthcare professionals to understand and trust the model’s predictions, aiding in preventive actions.

eXtreme Gradient Boosting (XGBoost) was used in four studies [46,51,57,69]. It provided accurate infection predictions by effectively managing large datasets and handling both classification and regression tasks. Its importance lied in enhancing prediction accuracy, particularly in identifying patterns that indicate a heightened risk of infection.

Weka was used in four studies [28,50,52,60]; Weka is a comprehensive Machine Learning software suite that was applied to build and validate various infection control models. Its diverse toolkit allow researchers to experiment with different algorithms and identify the most effective ones for predicting infection risks.

MOCA-I (Multi-Objective Classification Algorithm for Imbalanced Data) was employed in one study [41]. MOCA-I is specifically designed to address class imbalance, a common issue in healthcare data where infection cases might be far fewer than non-infection cases. Its use was critical in improving model performance for rare infection events, reducing false negatives, and improving sensitivity.

CalcAMP software was used in one study [29] that focused on antimicrobial peptide (AMP) prediction. CalcAMP applied AI to predict the efficacy of various AMPs in combating bacterial infections, aiding in the development of new therapeutic strategies to combat antibiotic resistance.

Bayesian Network (BN) was featured in three studies [27,42,68]. BN models were utilized for their ability to model probabilistic relationships between variables. They were applied to understand the complex interactions between patient characteristics and infection outcomes, allowing for the creation of predictive models that can adjust as new information is obtained.

Least Absolute Shrinkage and Selection Operator (LASSO) was used in one study [64]. LASSO is a regularization technique for regression that helps select the most relevant variables, reducing overfitting. In infection control, it played an important role in narrowing down the critical factors contributing to infection risk, enhancing the accuracy and reliability of predictive models.

MALDI BIOTYPER tool version 2.0 was used in conjunction with ClinProTools in one study [30]. It is a mass spectrometry-based identification system, widely used for microorganism identification. Its application in infection control studies focused on rapid pathogen identification, aiding in early detection and precise treatment interventions.

Gradient Boosting Machine (GBM) was featured in two studies [25,73]. GBM is a powerful ensemble Machine Learning method that sequentially builds models to correct errors from previous iterations. In infection control, GBM was used to predict infection risks by improving the accuracy of models with each iteration, making it ideal for handling imbalanced datasets, such as those often seen in healthcare.

##### Hybrid Models: Machine Learning (ML) and Deep Learning (DL)

Eight studies [25,32,40,51,55,65,66,73] employed hybrid models that combined Machine Learning and Deep Learning to increase the accuracy and adaptability of infection control predictions. These hybrid approaches were especially valuable in handling complex infection datasets that required a combination of classification, prediction, and pattern recognition.

SVMs and Artificial Neural Networks (ANNs) were used in three studies [22,25,35]. These combined methods improved predictive performance by leveraging both the classification power of SVMs and the pattern recognition strength of NNs. This was important for clinical studies that needed to predict infection spread based on diverse patient data. Ensemble methods, such as combining RF with DL models, were used in eight studies [22,24,25,35,45,47,49,73]. They improved the robustness of predictions, especially in detecting multidrug-resistant organisms (MDROs). These models reduced prediction errors by aggregating results from multiple algorithms, making them more reliable in clinical use.

ClinProTools software version 3.0 was used in one study [66]. ClinProTools is designed for mass spectrometry data analysis. In this study, the software was employed to analyze complex protein patterns related to infections, making it a critical tool for identifying biomarkers associated with infection risks and outcomes.

Long Short-Term Memory (LSTM) Neural Networks were used in two studies [51,73]. LSTM networks excel at processing sequences of data, making them particularly useful in tracking the temporal progression of infections. Their importance lies in their ability to model sequential data, such as patient vitals over time, which aids in predicting infection onset and progression in healthcare settings.

##### Deep Learning (DL)

Four studies exclusively employed Deep Learning (DL) algorithms [48,67,72,76], which were highly effective in handling large and complex infection datasets without the need for extensive feature engineering. Convolutional Neural Networks (CNNs) were used in two studies [48,51] and were crucial for interpreting medical imaging data to detect bacterial infections. Their Deep Learning capabilities enabled the automatic detection of complex patterns in images, which improved diagnostic accuracy and the early detection of infections. Graph Neural Networks (GNNs) were used in one study [76]. They modeled the relationships between patients and infection spread, especially within healthcare facilities. This was particularly important in understanding how infections propagate through networks of patients and staff, aiding in infection control measures.

YOLO v5 was applied in one study [72]. YOLO v5 (You Only Look Once) is a real-time object detection algorithm typically used in imaging. In the context of infection control, YOLO v5 was employed for detecting infection-related patterns in medical images. Its real-time processing capabilities are crucial for timely diagnostics, particularly in detecting infection markers or abnormalities in imaging data.

##### Computational Biology and Machine Learning

Three studies integrated Machine Learning with computational biology tools, which were critical for pathogen identification and advancing therapeutic research, especially in the academic context. BLASTPhage, BLASTHost, and CRISPRPred were used in one study [24]. It utilized alignment-based methods to identify and classify bacteriophages, a crucial step in developing phage therapy as an alternative to antibiotics. This tool was important in academic research for discovering potential phage candidates that could be used to treat antibiotic-resistant bacterial infections. The Knowledge-Based Bayesian Network (KBBN) was used in one study [26], where the KBBN combined domain knowledge with Bayesian inference to improve prediction accuracy in infection control. This model integrated clinical knowledge into its predictions, which was particularly valuable for complex scenarios where purely data-driven approaches might struggle.

##### Knowledge Discovery and Semantic Analysis (KD and SA)

One study [33] employed knowledge discovery and semantic analysis (KD and SA) techniques, which were vital for processing and interpreting large volumes of infection control data. Rule-based systems were used in one study [33]. It processed unstructured clinical data by applying predefined rules to derive actionable insights. Its importance lies in its ability to organize disparate data sources into meaningful information that could guide infection control protocols.

##### Machine Learning (ML), Deep Learning (DL), and Natural Language Processing (NLP)

One study [20] uniquely combined Machine Learning, Deep Learning, and Natural Language Processing (NLP) to analyze vast amounts of unstructured textual data, such as patient records and medical reports. word2vec and GloVe were used in one study [20]. These NLP models extracted key infection control insights from text data, enabling the identification of trends in infection spread and improving the accuracy of infection risk predictions. This combination of AI techniques was critical for processing non-standardized clinical data, providing deeper insights into infection dynamics.

#### 2.2.4. Advantages

Six categories of advantages were identified: enhanced diagnostic accuracy, cost-effectiveness and efficiency, improved treatment effectiveness, predictive modeling and risk assessment, early detection and prevention, and data utilization and integration. Among these, 28 studies focused on predictive modeling and risk assessment [22,23,24,27,29,31,34,35,36,37,41,42,45,46,49,51,52,54,55,56,57,59,62,63,67,68,73,76], 18 studies emphasized enhanced diagnostic accuracy [20,21,26,28,30,33,40,43,44,48,50,53,60,65,66,69,71,72], and 6 studies were centered on early detection and prevention [25,39,47,61,64,70]. Cost-effectiveness and efficiency [77], along with improved treatment effectiveness [32], each had one study with the respective focus.

#### 2.2.5. Limitations

The six identified categories for limitations included data limitation, lack of real-world validation, limited scope and applicability, complexity and time-consuming, generalizability issues, and technical and computational challenges. Among these, 36 studies reported limitations related to generalizability issues [22,23,25,27,28,29,31,33,34,35,36,37,40,41,42,44,45,46,47,49,53,54,56,57,59,60,63,65,67,68,69,70,71,73,76,77]; 10 studies cited a lack of real-world validation [20,21,32,39,48,51,55,61,64,72]; 5 studies mentioned limited scope and applicability [26,30,43,52,66]; 2 studies highlighted data limitations [24,50]; and 1 study noted technical and computational challenges [62]. None of the studies reported issues related to complexity and time consumption.

## 3. Discussion

This scoping review aimed to understand the characteristics of AI applications in bacterial infection control. The search from 3 databases yielded a total of 1165 articles, where only 54 articles met the eligibility criteria and were extracted and analyzed. Five thematic scopes were synthesized from the extracted data: countries, aim, type of AI, advantages, and limitations of AI applications in infection prevention and control. The Global Alliance for Infections in Surgery developed a position statement called Worldwide Antimicrobial Resistance National/International Network Group (WARNING); its main goal is to enhance the global antibiotic stewardship programs through clinical practice guidelines [78]. Artificial intelligence has an important role in antimicrobial resistance, outbreak surveillance, and infection control [79].

The extracted data showed no contribution from the low-income countries; the majority of AI in infection control contributions were reported from high-income countries, which reflects the need to improve the evidence-based guidance for the global infection prevention and control program. Villanueva 2022 marked a gap in IPC and antimicrobial stewardship between low- and high-income countries and called for a collaborative international approach to combat antimicrobial resistance [80]. Another study discussed the challenges faced to apply IPC in limited resources countries, with the most relative being the lack of infrastructure and political commitment, which are key aspects of AI [81]. Another systematic review discussed the barriers to utilize AI in healthcare in middle and low resources countries, which include limited data availability and cost-effectiveness [82].

Our scoping review sheds light on important concepts of infection control including surgical site infection, healthcare-associated infection, and septicemia. These are the most common areas that require effective risk assessment as these risk factors are linked to mortality [83]. The reported bacterial strains that were involved AI in their identification, diagnosis, and treatment were *Staphylococcus aureus*, *Klebsiella* spp., *Pseudomonas aeruginosa*, *Enterococcus*, *Clostridium difficile*, *Mycobacterium tuberculosis*, other MDROs, VRE, and Carbapenem-resistant Gram-negative bacilli. The majority are members of ESKAPE pathogens that are commonly known for their resistance pattern [84,85,86,87]. These pathogens contribute to the increasing burden of antimicrobial resistance due to their tendency for genetic mutation and the acquisition of mobile genetic elements (MGEs) that helped in developing resistance against certain antibiotic classes [84]. This is consistent with the species indicated on the WHO’s 2022 Global Antimicrobial Resistance and Use Surveillance System (GLASS) report [88].

The majority of AI applications used in clinical settings were dominated by Machine Learning algorithms (ML), such as Logistic Regression (LR), Random Forest (RF), Support Vector Machines (SVMs), and Decision Trees (DTs). These models were primarily applied to pathogen identification, early infection detection, and risk assessment of healthcare-associated infections (HAIs). This is consistent with the design of these AI models, which excel at predictive modeling, classification, and decision support, making them ideal for real-time clinical use. For instance, in this scoping review, AMRQuest software v.2.1 was used to perform the presumptive identification of *Methicillin-resistant Staphylococcus aureus* (MRSA) [21]. This model showcased the high diagnostic accuracy of ML algorithms in detecting antibiotic-resistant pathogens, highlighting AI’s crucial role in infection control within healthcare settings. These results go hand in hand with Airlangga G 2024 [89], who reported the effectiveness and robustness of LR and RF in infection prediction and diagnostics, demonstrating a significant improvement over standard diagnostic practice. Furthermore, another recent study by Yang 2024 [90] demonstrated the ability to rapidly detect bacterial toxins using ML-enhanced Raman spectroscopy. This solidifies the role of the various AI-enabled applications in infection control with the potential to enhance diagnostic accuracy, streamline workflows, and reduce the spread of infectious diseases.

Some studies integrated hybrid AI approaches to enhance the robustness of their models. For example, in this scoping review, one study used hybrid Machine Learning and Deep Learning (DL) models, such as SVM and Neural Networks, to predict excess growth in genotyped tuberculosis [25]. These hybrid approaches allowed for more nuanced and adaptable predictive capabilities, particularly in complex clinical scenarios. This is consistent with findings by Ghaffar 2023 [91], who reported that combining ML and DL in hybrid models improves the performance of AI systems in diverse healthcare applications, especially in infection control and monitoring. On the other side, Rahman 2024 pointed the real challenges that come with integrating hybrid AI approaches especially ML and DL in healthcare prediction and infection control [92]. The complexity of Deep Learning (DL) models exacerbates the issue. DL models, due to their multiple layers, tend to be large and computationally intensive. This creates significant hurdles when trying to integrate these models into environments such as the Internet of Things (IoT) and wireless sensor networks, where resources are often constrained, making deployment more difficult [93]. A recent study utilized a novel Deep Learning model which combined a pre-trained CNN (ChexNet) encoder with a Self-MLP classifier, showing enhanced efficacy of diagnostics (in detecting infectious disease such as tuberculosis) [94].

Some studies utilized sophisticated AI software like L2-regularized Logistic Regression models to improve clinical decision-making in infection control. In this scoping review, one study applied this model to assess patient risk for hospital-onset *Clostridioides difficile* infections (CDIs), a critical pathogen in healthcare-associated infections. The regularization technique used in this model helped prevent overfitting, ensuring that predictions remained generalizable across different patient populations [23]. This consistent use of advanced AI methods, such as those by Goswami 2024, emphasized the importance of algorithmic optimization techniques like regularization in enhancing the accuracy and applicability of predictive models in healthcare datasets [95].

The integration of AI in bacterial infection control presents significant ethical considerations that must be addressed to ensure responsible implementation. One primary concern is bias in AI models, which can stem from imbalanced or non-representative datasets, potentially leading to disparities in infection risk assessment and treatment recommendations. If AI systems are trained predominantly on data from high-income settings, their applicability to low resource environments may be limited, exacerbating existing healthcare inequalities [96,97]. Another critical issue is data security and patient privacy. AI models rely on vast amounts of healthcare data, raising concerns about unauthorized access, data breaches, and compliance with regulations such as General Data Protection Regulation (GDPR) and the Health Insurance Portability and Accountability Act (HIPAA). Ensuring robust encryption, secure data-sharing protocols, and stringent access controls is essential to maintaining patient confidentiality while leveraging AI for infection control [98,99]. Furthermore, AI-driven decision-making introduces ethical challenges regarding accountability and transparency. When AI systems provide recommendations for infection management, it is crucial to establish clear guidelines on human oversight and responsibility. Clinicians must understand the reasoning behind AI-generated outputs to make informed decisions, minimizing the risk of automation bias and ensuring ethical application in clinical practice [100,101].

Overall, the diverse AI software and algorithms used reflect their importance in achieving the goals of studies. In clinical settings, AI helps reduce the burden of HAIs by providing accurate, real-time predictions and diagnostics, and drives forward research into novel infection control methods and therapeutic strategies. These consistent findings underscore the transformative potential of AI across various domains of infection control, aligning with the objectives and anticipated outcomes of the scoping review. The integration of advanced AI systems, whether through Machine Learning, Deep Learning, or hybrid approaches, plays a critical role in enhancing the effectiveness of infection prevention and control efforts, ultimately contributing to improved public health outcomes globally.

The major advantages of using AI in infection control are predictive modeling and risk assessment that directly contribute to clinical decision-making [102,103]. Cost-effectiveness and efficiency are also two important concepts; however, there is insufficient data to conclude the cost-effectiveness and efficiency of using AI in infection control [104]. The most notable limitation was generalizability and this was reported in several other studies; when the model is built using data from specific institution, it might not be applicable on other medical facilities and therefore training the model on data from different sources becomes necessary to enhance the generalizability [105]. In addition, one more limitation is the utility of AI in low-income countries. The noted deficiency in AI applicability in low-income countries could be addressed via fostering international collaborations, building local AI infrastructure, and providing targeted training for healthcare workers in low-income countries [106].

This scoping review has some limitations such as the absence of Risk of Bias Assessment (RoB) of the extracted studies due to differences in the study designs. Another limitation is subject variability and complexity of the concepts reported in the articles, which limited the capacity to extract information from the studies. However, this scoping review is a landmark toward other studies and systematic reviews to further understand AI in infection control. The findings of this study contribute to enhancing the public health strategies that tackle infectious diseases and promote antimicrobial stewardship efforts.

## 4. Materials and Methods

This scoping review was conducted according to Arksey and O’Malley frameworks. We mapped the key concepts of AI research in infection control by summarizing and synthesizing the available sources of evidence. This study is a precursor of a forthcoming systematic review, in which we assessed the extent and nature of published research on this topic. We adhered to the stages outlined by Arksey and O’Malley [107] and the recommendations by Levac [108]. The stages included developing the following research question: what are the characteristics of AI in bacterial infection control? Relevant studies were identified by creating well-defined search strategies across three databases and evaluating their relevance. Study selection was carried out based on specific eligibility criteria. Qualitative data charting was performed using a detailed extraction sheet, following the recommendations of Ritchie and Spencer [109,110] (Supplementary: S6). Data collection and synthesis included keyword coding and thematic scopes analysis, conducted in consultation with experts in bacterial infection control, resulting in the identification of six thematic scopes according to the reported quantitative data.

### 4.1. Data Sources and Search Strategy

We conducted comprehensive searches of three databases: PubMed, Embase, and Web of Science. Our searches, completed on 9 June 2024, utilized broad search terms in titles and abstracts incorporating MeSH and Emtree terms expanded to include all subheadings, titles, and free-text terms. We used the following keywords to conduct our search: “machine learning”, “computational intelligence”, “computer reasoning”, “computer vision system”, “knowledge acquisition,”, “knowledge representation”, “deep learning”, “neural network”, “machine intelligence”, “artificial intelligence”, “Infection Control”, “infection prevention”, “contamination control”, “infection management”, “biosecurity measures”, “sanitation practices”, “hygiene control”, “pathogen control”, “epidemic control”, “bacteria s”, “bacteriae”, “bacterias”, “microbiology”, “bacteria”, “pathogen”, “microorganism”, “germ”, and “microbe” (Supplementary: S1).

The search strategy was re-run before the thematic analyses to retrieve the new publications. A bibliography search was conducted to ensure that no relevant publications on this topic were overlooked.

The citations obtained from our search strategy were imported to EndNote citation management software (v20.2.1). All team members contributed to the development of the search strategy, which was created in accordance with the Peer Review of Electronic Search Strategies (PRESS) 2015 checklist (Supplementary: S2) [111,112].

### 4.2. Study Selection and Eligibility Criteria

Eligibility criteria included all publications reporting the applications of AI in bacterial infection prevention and control. Studies must explore the utilization and effectiveness of AI in diagnostics, prevention, or outbreak investigation either within in-hospital or community-based settings. Furthermore, studies about AMR prevention using AI were included. Exclusions were made for studies investigating non-bacterial infections and studies that do not report AI performance indicators. Publications that were not based on primary data, case reports, case series, editorials, commentaries, reviews, conference abstracts, and non-peer-reviewed e-print archives were also excluded.

### 4.3. Data Extraction and Synthesis

Citations from the search strategies were imported into EndNote (version 20.2.1) for screening, and duplicates were identified and removed using the reference manager tool. The first screening of titles and abstracts was conducted by three authors using the rayyan platform, classifying citations as relevant, potentially relevant, or not relevant. Four independent authors then rescreened, extracted, and double-extracted the citations. An assessor author reviewed and validated the double-extracted data. Discrepancies were resolved through discussion with the corresponding author. All data extraction and synthesis were conducted on Microsoft Excel.

### 4.4. Standardization

This scoping review followed the Preferred Reporting Items for Systematic Reviews and Meta-Analyses extension for Scoping Reviews (PRISMA-ScR) checklist (Supplementary: S2) [113]. The protocol was registered in Open Science Framework (OSF) on 9 November 2024 and can be accessed through the following link: https://doi.org/10.17605/OSF.IO/R25KW [114].

### 4.5. Data Cleaning and Handling Ambiguous and Missing Data

Ambiguous data were addressed through discussions among the research team with efforts to contact the original authors for clarification. Unresolved missing information was noted in the analysis, ensuring transparency in the research findings while acknowledging limitations due to unavailable data. Data cleaning was performed by four authors, involving the summarization and restructuring of data using keywords and categories to generate the thematic scopes. Categorization was conducted for the advantages and limitations of AI tools, resulting in organized and standardized data for analysis.

### 4.6. Data Synthesis and Generation of Thematic Scopes

Data underwent comprehensive narrative mapping and categorization, followed by the construction of five thematic scopes: countries, aim, type of AI, advantages, and limitations. The three thematic scopes of aim, advantages, and limitations involved coding keywords and the generation of categories that helped in mapping and summarizing the extracted articles (Supplementary: S3, S4, S5). Those categories reflect shared characteristics of the extracted articles. Coding, categorizing, and data synthesis were performed collaboratively, with researchers working in pairs, and uncertainties were resolved through group discussions.

## 5. Conclusions

This study aimed to explore AI applications in infection control, with an initial search revealing no similar studies or review protocols. The review of 54 studies highlighted the effectiveness of AI models for bacterial infection control, particularly in predictive modeling, risk assessment, and diagnostic accuracy, with Machine Learning algorithms like Logistic Regression, Random Forest, and SVM being widely used. However, the study also identified significant limitations, including generalizability issues, lack of real-world validation, and data constraints, which hinder broader applicability. In addition, the under representation of AI utilization in low-income countries can be tackled by international collaborations and unique training programs. The findings emphasize AI’s potential in infection control while recognizing the need to address these challenges to enhance its implementation and impact in diverse clinical settings.

## Figures and Tables

**Table 1:** Studies’ characteristics including citation, country, name of AI, type of AI, summarized aim, scope of aim, scope of advantages, and scope of limitations.

Author’s Name	Country	Name of AI	Type of AI	Summarized Aim	Scope of Aim	Advantages	Limitations
Jeon K, 2022 [21]	Korea	AMRQuest software, v.2.1	Machine Learning	Presumptive identification of MRSA	Pathogen identification	Enhanced Diagnostic Accuracy	Lack of Real-World Validation
Çaǧlayan Ç, 2022 [22]	USA	Logistic regression (LR), random forest (RF), extreme gradient boosting (XGBoost), artificial neural network (ANN), support vector machine (SVM)	Machine Learning	Identify patients likely to be colonized with VRE, CRE, or MRSA upon ICU admission	Pathogen identification	Predictive Modeling and Risk Assessment	Generalizability Issues
Ötleş E, 2023 [23]	USA	L2-regularized logistic regression model	Machine Learning	Assess patient risk for hospital-onset CDI and evaluate effectiveness of AI models	Pathogen identification	Predictive Modeling and Risk Assessment	Generalizability Issues
Aggarwal S, 2023 [24]	India	Alignment-based methods: BLASTPhage, BLASTHost, CRISPRPred Machine learning models: Random Forest (RF) and Gaussian Naive Bayes (GNB) Hybrid model: Ensemble method:	Machine Learning and Computational Biology	Facilitate researchers in the field of phage therapy	Therapeutic	Predictive Modeling and Risk Assessment	Data Limitation
Althomsons S, 2022 [25]	USA	ML/DL techniques after NBH (DT, RF, SVM, Regularized regression, Ensemble methods, GBM, ANNs)	Machine Learning and Deep Learning	Predict excess growth in genotyped tuberculosis clusters, with the goal of early identification of clusters	Pathogen identification	Early Detection and Prevention	Generalizability Issues
Aminian M, 2014 [26]	USA, France	Knowledge-based Bayesian network (KBBN)	Machine Learning and Computational Biology	Improve the classification accuracy of *Mycobacterium tuberculosis* complex (MTBC) clades	Pathogen identification	Enhanced Diagnostic Accuracy	Limited Scope and Applicability
Atkinson A, 2023 [27]	Switzerland	Decision trees, and Network graph analysis	Machine Learning	Improve existing outbreak investigation processes	Outbreak investigation and surveillance	Predictive Modeling and Risk Assessment	Generalizability Issues
Azé J, 2015 [28]	Netherlands, Pakistan, France	Weka	Machine Learning	Develop a consensual taxonomy for MTC	Pathogen identification	Enhanced Diagnostic Accuracy	Generalizability Issues
Bournez C, 2023 [29]	Switzerland	CalcAMP	Machine Learning	Accelerate the discovery of new AMPs as alternatives to antibiotics	Therapeutic	Predictive Modeling and Risk Assessment	Generalizability Issues
Camoez M, 2016 [30]	Spain	CLINPROTOOLS, MALDI BIOTYPER	Machine Learning	Automated discrimination of major MRSA lineages and to develop a reliable tool for S. aureus typing	Pathogen identification	Enhanced Diagnostic Accuracy	Limited Scope and Applicability
Cheah, A. L. Y, 2018 [31]	Australia	Hidden Markov Model (HMM) in conjunction with Bayesian inference	Machine Learning	Effectively control VRE spread in healthcare settings	Outbreak investigation and surveillance	Predictive Modeling and Risk Assessment	Generalizability Issues
Cherkasov A, 2009 [32]	Canada	Artificial Neural Networks (ANNs)	Machine Learning and Deep Learning	Asses the antibacterial, physical, and harmful properties of a variety of small peptide antibiotics	Therapeutic	Improved Treatment Effectiveness	Lack of Real-World Validation
de Bruin JS, 2017 [33]	Austria	Rule-based system for processing medical knowledge, which is more related to knowledge representation and reasoning in the field of artificial intelligence.	Knowledge Discovery and Semantic Analysis	Facilitating electronic HAI surveillance	Infection risk assessment	Enhanced Diagnostic Accuracy	Generalizability Issues
Doan, T. N, 2015 [34]	Australia	Hidden Markov models (HMMs)	Machine Learning	Characterize the transmission dynamics of *Acinetobacter baumannii* in ICUs	Pathogen identification	Predictive Modeling and Risk Assessment	Generalizability Issues
Feng C, 2023 [35]	China	Artificial Neural Network (ANN), Support Vector Machine (SVM), Logistic Regression, Random Forest, K-Nearest Neighbor, Decision Tree, and XGBoost.	Machine Learning	Predicting invasive *Klebsiella pneumoniae* liver abscess syndrome (IKPLAS) in diabetes mellitus	Pathogen identification	Predictive Modeling and Risk Assessment	Generalizability Issues
Freire, M. P, 2022 [36]	Brazil, Italy	Random Forest Classifier	Machine Learning	Predict CRE colonization	Pathogen identification	Predictive Modeling and Risk Assessment	Generalizability Issues
Goodman, K. E, 2019 [37]	USA	Decision trees (DT); classification and regression tree (CART) algorithm, “rpart” package, version 4.1–13, was used in the R statistical package (version 3.0.5)	Machine Learning	Predict the probability of CRO and/or CPO carriage	Pathogen identification	Predictive Modeling and Risk Assessment	Generalizability Issues
Gouareb R, 2023 [38]	Switzerland	Graph Neural Networks (GNNs)	Deep Learning	Predict the risk of inpatient colonization by MDR Enterobacteriaceae	Pathogen identification	Predictive Modeling and Risk Assessment	Generalizability Issues
Hattori S, 2020 [39]	Japan	Rotation Forest ensembles in Weka	Machine Learning	Early identification of clinically important bacteria	Pathogen identification	Early Detection and Prevention	Lack of Real-World Validation
Hsu, C. C, 2008 [40]	USA, Taiwan	Artificial Neural Networks (ANNs)	Machine Learning and Deep Learning	Create a tool that can aid in infection control and potentially reduce the need for active surveillance cultures, which are costly and labor-intensive	Pathogen identification	Enhanced Diagnostic Accuracy	Generalizability Issues
Jacques J, 2020 [41]	France	Multi-Objective Classification Algorithm for Imbalanced data (MOCA-I)	Machine Learning	Identify a set of risk factors for MDR pathogen carriage and infection.	Pathogen identification	Predictive Modeling and Risk Assessment	Generalizability Issues
Jakobsen, R. S, 2024 [42]	Denmark	Bayesian Network (BN)	Machine Learning	Risk stratification of hospital-acquired urinary tract infections (HA-UTI)	Infection risk assessment	Predictive Modeling and Risk Assessment	Generalizability Issues
Khaledi A, 2016 [43]	Germany	Potential Support Vector Machine (P-SVM)	Machine Learning	Genome based ML detection of resistance in *Pseudomonas aeruginosa*	Antimicrobial resistance and stewardship	Enhanced Diagnostic Accuracy	Limited Scope and Applicability
Khaledi A, 2020 [44]	Germany, Spain, Hungry, Romania	SVM	Machine Learning	Predictive models and identified biomarkers of resistance to four commonly administered antimicrobial drugs	Antimicrobial resistance and stewardship	Enhanced Diagnostic Accuracy	Generalizability Issues
Lapp Z, 2021 [45]	USA	SVM with a radial basis kernel, L2 regularized logistic regression, Elastic net, Random Forest	Machine Learning	Understand which factors, whether patient-related or microbial genomic, could discriminate between CRKP extraintestinal colonization and infection across multiple healthcare facilities	Pathogen identification	Predictive Modeling and Risk Assessment	Generalizability Issues
Liang, Q. Q, 2024 [46]	China	XGBoost, SVM, Random Forest	Machine Learning	Predicting the occurrence of bloodstream infection and associated factors	Pathogen identification	Predictive Modeling and Risk Assessment	Generalizability Issues
Liang, Q. Q, 2022 [47]	China	Random forest, XGBoost, Decision tree, Multivariable logistic regression	Machine Learning	Predict the occurrence of CR-GNB carriage in Intensive Care Unit (ICU) patients	Pathogen identification	Early Detection and Prevention	Generalizability Issues
Lyu, J. W, 2023 [48]	China	Convolutional Neural Network (CNN)	Deep Learning	Prediction of multidrug-resistant *K. pneumoniae*	Pathogen identification	Enhanced Diagnostic Accuracy	Lack of Real-World Validation
Marra, A. R., 2020 [49]	USA	SVM, decision trees, multilayer perceptron, radial basis function classifiers, K-nearest neighbor, bagging, boosting, logistic regression, random forest, and naïve Bayes models.	Machine Learning	Predict *Clostridioides difficile* infection in hospitalized patients using routinely available clinical data	Pathogen identification	Predictive Modeling and Risk Assessment	Generalizability Issues
Noman, S. M., 2023 [50]	65 countries	Weka (v3.9.2), Java	Machine Learning	Enhance the accuracy of antimicrobial resistance predictions	Antimicrobial resistance and stewardship	Enhanced Diagnostic Accuracy	Data Limitation
Panchavati, S., 2022 [51]	USA	XGBoost, Deep Long Short Term Memory neural network (D-LSTM), and one-dimensional convolutional neural network (1D-CNN)	Machine Learning and Deep Learning	Predict *Clostridioides difficile* infection (CDI) in hospitalized patients, facilitate enhanced clinical monitoring, earlier diagnosis, and timely implementation of infection control measures	Pathogen identification	Predictive Modeling and Risk Assessment	Lack of Real-World Validation
Rabhi, S., 2018 [20]	France	word2vec, Glove	Machine Learning and Deep Learning and Natural Language Processing	Detecting healthcare-associated infections (HAIs), to determine which method provides better accuracy and reliability in classifying HAIs using textual electronic medical records	Infection risk assessment	Enhanced Diagnostic Accuracy	Lack of Real-World Validation (Opacity of CNNs)
Ratzinger, F., 2015 [52]	Austria	Weka, R, MDCalc bvba	Machine Learning	Determine whether routine laboratory parameters could be used as surrogate markers to predict the type of bacterial pathogen in bloodstream infections	Infection risk assessment	Predictive Modeling and Risk Assessment	Limited Scope and Applicability
Rennert-May, E., 2022 [53]	Canada	Python version 3.9.12 and Scikit-Learn (used to train the logistic regression models)	Machine Learning	Determine the best approach for identifying CIED infections	Infection risk assessment	Enhanced Diagnostic Accuracy	Generalizability Issues
Rhodes, N. J., 2023 [54]	USA	Optimal data analysis	Machine Learning	Predict the risk of Methicillin-resistant *Staphylococcus aureus* (MRSA) in hospitalized patients with community-acquired pneumonia (CAP) early in the course of hospital admission	Pathogen identification	Predictive Modeling and Risk Assessment	Generalizability Issues
Sambarey, A., 2024 [55]	Multiple countries	Python v. 3.7.14, Matlab R2021b, R studio v.4.3.0	Machine Learning and Deep Learning	Improve the prediction of treatment outcomes and guide personalized treatment strategies for TB, particularly in the context of drug-resistant TB	Therapeutic	Predictive Modeling and Risk Assessment	Lack of Real-World Validation
Savin, I., 2018 [56]	Russia	RF and XGBoost	Machine Learning	Determine the incidence of healthcare-associated ventriculitis and meningitis (HAVM) in a neuro-ICU	Infection risk assessment	Predictive Modeling and Risk Assessment	Generalizability issues
Schinkel, M., 2022 [57]	USA, Netherlands	XGBoost	Machine Learning	Predict blood culture outcomes in the emergency department	Infection risk assessment	Predictive Modeling and Risk Assessment	Generalizability Issues
Seheult, J. N., 2023 [58]	USA	Software Represented Using AI: R v 3.4.2 (with the “rpart” package): The machine learning decision tree algorithm (PittUDT) was implemented using the “rpart” package in R. (R v 3.4.2 itself is not AI)	Machine Learning	Optimize urinalysis parameters for predicting urine culture positivity	Infection risk assessment	Cost-Effectiveness and Efficiency	Generalizability Issues
Shohat, N., 2020 [59]	USA, Europe	Random Forest (RF)	Machine Learning	Accurately predict the outcome following irrigation and debridement (I&D) surgery for prosthetic joint infection	Infection risk assessment	Predictive Modeling and Risk Assessment	Generalizability Issues
Singh, H., 2023 [60]	USA	Weka (version 3.8.6)	Machine Learning	Identify predictive biomarkers for latent *Mycobacterium tuberculosis* infection	Pathogen identification	Enhanced Diagnostic Accuracy	Generalizability Issues
Sundermann, A. J., 2021 [61]	USA	Enhanced Detection System for Healthcare-Associated Transmission (EDS-HAT)	Machine Learning	Enhance outbreak detection in hospitals by combining whole genome sequencing (WGS) surveillance, to identify and trace transmission routes of healthcare-associated infections	Outbreak investigation and surveillance	Early Detection and Prevention	Lack of Real-World Validation
Tacconelli, E., 2020 [62]	Italy, Serbia, Romania	Random Forest (RF) algorithm	Machine Learning	Measure the impact of antibiotic exposure on the acquisition of colonization with extended-spectrum β-lactamase-producing Gram-negative bacteria	Therapeutic	Predictive Modeling and Risk Assessment	Technical and Computational Challenges
Tadesse, B. T., 2023 [63]	Bangladesh	R Studio analytical software (R Foundation for Statistical Computing) “rpart” for decision tree modeling, “rpart.plot” for tree plotting, “pROC” for ROC curve analysis, “survival” for Cox models, and “dplyr” for data management	Machine Learning	Assess the association between household WASH status and typhoid risk in urban slums	Infection risk assessment	Predictive Modeling and Risk Assessment	Generalizability Issues
Tsurumi, A, 2023 [64]	USA	Least Absolute Shrinkage and Selection Operator (LASSO) “machine learning AI algorithm”	Machine Learning	Predicting bloodstream infections in children with burns	Infection risk assessment	Early Detection and Prevention	Lack of Real-World Validation
Wang, H. Y. 2018 [65]	Taiwan	Decision tree (DT), Support vector machine (SVM), and k-nearest neighbor (KNN) for predictive modeling	Machine Learning and Deep Learning	Develop a new scheme for strain typing of methicillin-resistant *Staphylococcus aureus* (MRSA)	Pathogen identification	Enhanced Diagnostic Accuracy	Generalizability Issues
Wang, H. Y, 2018 [66]	Taiwan	ClinProTools software version 3.0	Machine Learning and Deep Learning	Classifying major MLST types of MRSA	Pathogen identification	Enhanced Diagnostic Accuracy	Limited Scope and Applicability
Wang, Y, 2023 [67]	China	Backpropagation Neural Network (BPNN)	Deep Learning	Predicting multidrug-resistant organism (MDRO) infection in critically ill patients	Pathogen identification	Predictive Modeling and Risk Assessment	Generalizability Issues
Waterhouse, M, 2011 [68]	Australia	Bayesian Networks (implemented in Netica and WinBUGS softwares utilizing AI)	Machine Learning	Understand the complex system of interrelationships between various factors that affect this transmission	Infection risk assessment	Predictive Modeling and Risk Assessment	Generalizability Issues
Wu, G, 2023 [69]	Canada	XGBoost	Machine Learning	Automated detection of complex surgical site infections (SSIs) following total hip and knee arthroplasty	Infection risk assessment	Enhanced Diagnostic Accuracy	Generalizability Issues
Yan, M, 2022 [70]	China	Markov Model (MM): Machine Learning / Computational Biology Neural Network (NN): Machine Learning Support Vector Machine (SVM): Machine Learning Integrated Promoter Markov Discriminant (IPMD) algorithm: Machine Learning / Computational Biology	Machine Learning and Computational Biology	Establish an early warning system for the epidemic mechanism	Outbreak investigation and surveillance	Early Detection and Prevention	Generalizability Issues
Zeng, Z, 2024 [71]	China	Nested Logistic Regression Models (classified under Machine Learning (ML) rather than being standalone AI)	Machine Learning	Accurately classify pulmonary status caused by *Acinetobacter baumannii*	Pathogen identification	Enhanced Diagnostic Accuracy	Generalizability Issues
Zhong, Z, 2022 [72]	China	YOLO v5	Deep Learning	Diagnostic accuracy of AI models in identifying the *Helicobacter pylori*	Pathogen identification	Enhanced Diagnostic Accuracy	Lack of Real-World Validation
Zwerwer, L. R, 2024 [73]	Netherlands	Long Short-Term Memory (LSTM) neural networks, Gradient Boosting Machines, Random Forest, Logistic Regression	Machine Learning and Deep Learning	Predict the need for infection-related consultations in ICU patients	Therapeutic	Predictive Modeling and Risk Assessment	Generalizability Issues

**Table 2:** Matrix of thematic scopes.

Scope (1)	Countries	Low-Income			Middle-Income			High-Income		
**Scope (2)**	Aim	Pathogen Identification	Methicillin-Resistant *S. aureus*	*M. tuberculosis*	*Klebsiella* spp.	*C. difficile*	*A. baumannii*	Carbapenem-Resistant Gram-Negative	Multidrug-Resistant Organisms	Other
		Infection Risk Assessment		Healthcare-Associated Infections	Septicemia			Surgical Site Infection	Other Infections	
		Therapeutic								
		Outbreak Investigation and Surveillance								
		Antimicrobial Resistance Stewardship								
**Scope (3)**	Type of AI	Machine Learning	Hybrid	Deep Learning	Computational Biology and Machine Learning		Knowledge Discovery and Semantic Analysis	Machine Learning (ML), Deep Learning (DL), and Natural Language Processing (NLP)		
**Scope (4)**	Advantages	Enhanced Diagnostic Accuracy	Improved Treatment Effectiveness		Early Detection and Prevention		Cost-Effectiveness and Efficiency	Predictive Modeling and Risk Assessment		
**Scope (5)**	Limitations	Generalizability Issues	Lack of Real-World Validation		Limited Scope and Applicability		Technical and Computational Challenges	Data Limitation		

## Data Availability

The original contributions presented in this study are included in the article/Supplementary Materials. Further inquiries can be directed to the corresponding author.

## Supplementary Materials

The following supporting information can be downloaded at: https://www.mdpi.com/article/10.3390/antibiotics14030256/s1.

## Abbreviations

The following abbreviations are used in this manuscript:

IPC	infection prevention and control
AMR	antimicrobial resistance
HAIs	healthcare-associated infections
ECDC	European Centre for Disease Prevention and Control
WHO	World Health Organization
CAIs	community-associated infections
AI	artificial intelligence
ML	Machine Learning
DL	Deep Learning
LR	Logistic Regression
RF	Random Forest
XGBoost	Extreme Gradient Boosting
ANN	Artificial Neural Network
SVM	Support Vector Machine
CB	Computational Biology
KBBN	Knowledge-Based Bayesian Network
HMM	hidden Markov model
KD&SA	Knowledge Discovery and Semantic Analysis
DTs	Decision Trees
CART	Classification and Regression Tree
GNNs	Graph Neural Networks
MOCA-I	Multi-Objective Classification Algorithm for Imbalanced Data
BN	Bayesian Network
HA-UTIs	hospital-acquired urinary tract infections
ICU	Intensive Care Unit
CNN	Convolutional Neural Network
D-LSTM	Deep Long Short-Term Memory Neural Network
1D-CNN	One-Dimensional Convolutional Neural Network
CDI	Clostridioides Difficile Infection
NLP	Natural Language Processing
HAVM	healthcare-associated ventriculitis and meningitis
EDS-HAT	Enhanced Detection System for Healthcare-Associated Transmission
LASSO	Least Absolute Shrinkage and Selection Operator
KNN	K-nearest neighbor
MRSA	Methicillin-Resistant Staphylococcus Aureus
BPNN	Backpropagation Neural Network
MDRO	multidrug-resistant organism
SSIs	surgical site infections
NN	Neural Network
IPMD	Integrated Promoter Markov Discriminant
LSTM	Long Short-Term Memory
CAP	community-acquired pneumonia
VRE	Vancomycin-Resistant Enterococci
CRE	Carbapenem-Resistant Enterobacterales
EHR	Electronic Health Record
MTBC	Mycobacterium Tuberculosis Complex
MTC	Mycobacterium Tuberculosis Complex
LTBI	Latent TB
IKPLAS	Invasive Klebsiella Pneumoniae Liver Abscess Syndrome
DM	diabetes mellitus
CRKP	Carbapenem-Resistant Klebsiella Pneumoniae
MDR	multidrug-resistant
HO-CDI	hospital-onset Clostridioides difficile infection
CR-GNB	Carbapenem-Resistant Gram-Negative Bacterial Bloodstream
CROs	Carbapenem-Resistant Organisms
CPOs	Carbapenemase-Producing Organisms
HPCF	Helicobacter Pylori Coccoid Form
WGS	whole genome sequencing
AMP	antimicrobial peptide
GBMs	Gradient Boosting Machines
WARNING	Worldwide Antimicrobial Resistance National/International Network Group
MGEs	Mobile Genetic Elements
GLASS	Global Antimicrobial Resistance and Use Surveillance System
IoT	Internet of Things
RoB	Risk of Bias Assessment
OSF	Open Science Framework
PRISMA-ScR	Preferred Reporting Items for Systematic Reviews and Meta-Analyses Extension for Scoping Reviews
