# Supplementary material for: Artificial Intelligence in Bacterial Infections Control: A Scoping Review
Source: Antibiotics (Basel). 2025 Mar 2;14(3):256. doi: 10.3390/antibiotics14030256 (PMC11939793; doi:10.3390/antibiotics14030256)
Supplement: Supplementary file 1 [file antibiotics-14-00256-s001.zip › Suuplementary (S1-S5).pdf]

Supplementary

S1: Search strategy

PubMed

Search Details	Results
("machine learning"[All Fields] OR "computational intelligence"[All Fields] OR "computer reasoning"[All Fields] OR "computer vision system"[All Fields] OR "knowledge acquisition"[All Fields] OR "knowledge representation"[All Fields] OR "deep learning"[All Fields] OR "neural network"[All Fields] OR "machine intelligence"[All Fields] OR "artificial intelligence"[All Fields]) AND ("Infection Control"[All Fields] OR "infection prevention"[All Fields] OR "contamination control"[All Fields] OR "infection management"[All Fields] OR "biosecurity measures"[All Fields] OR "sanitation practices"[All Fields] OR "hygiene control"[All Fields] OR "pathogen control"[All Fields] OR "epidemic control"[All Fields]) AND ("bacteria s"[All Fields] OR "bacteriae"[All Fields] OR "bacterias"[All Fields] OR "microbiology"[MeSH Subheading] OR "microbiology"[All Fields] OR "bacteria"[All Fields] OR "bacteria"[MeSH Terms] OR "bacteria"[MeSH Terms] OR "pathogen*"[All Fields] OR "microorganism*"[All Fields] OR "germ*"[All Fields] OR "microbe*"[All Fields])	195
"Infection Control"[All Fields] OR "infection prevention"[All Fields] OR "contamination control"[All Fields] OR "infection management"[All Fields] OR "biosecurity measures"[All Fields] OR "sanitation practices"[All Fields] OR "hygiene control"[All Fields] OR "pathogen control"[All Fields] OR "epidemic control"[All Fields]	115,889
"epidemic control"[All Fields]	1,263
"pathogen control"[All Fields]	1,017
"hygiene control"[All Fields]	228
"sanitation practices"[All Fields]	330
"biosecurity measures"[All Fields]	1,086
"infection management"[All Fields]	2,228
"contamination control"[All Fields]	625
"infection prevention"[All Fields]	57,904
"Infection Control"[All Fields]	66,443
"machine learning"[All Fields] OR "computational intelligence"[All Fields] OR "computer reasoning"[All Fields] OR "computer vision system"[All Fields] OR "knowledge acquisition"[All Fields] OR "knowledge representation"[All Fields] OR "deep learning"[All Fields] OR "neural network"[All Fields] OR "machine intelligence"[All Fields] OR "artificial intelligence"[All Fields]	286,668
"artificial intelligence"[All Fields]	95,632
"machine intelligence"[All Fields]	1,734
"neural network"[All Fields]	78,642
"deep learning"[All Fields]	66,863
"knowledge representation"[All Fields]	1,177
"knowledge acquisition"[All Fields]	2,580
"computer vision system"[All Fields]	252
"computer reasoning"[All Fields]	8
"computational intelligence"[All Fields]	1,598
"machine learning"[All Fields]	124,637

"bacteria s"[All Fields] OR "bacteriae"[All Fields] OR "bacterias"[All Fields] OR "microbiology"[MeSH Subheading] OR "microbiology"[All Fields] OR "bacteria"[All Fields] OR "bacteria"[MeSH Terms] OR "bacteria"[MeSH Terms] OR "pathogen*"[All Fields] OR "microorganism*"[All Fields] OR "germ*"[All Fields] OR "microbe*"[All Fields]	5,985,976
"microbe*"[All Fields]	101,017
"germ*"[All Fields]	2,592,024
"microorganism*"[All Fields]	178,054
"pathogen*"[All Fields]	1,369,711
"bacteria s"[All Fields] OR "bacteriae"[All Fields] OR "bacterias"[All Fields] OR "microbiology"[MeSH Subheading] OR "microbiology"[All Fields] OR "bacteria"[All Fields] OR "bacteria"[MeSH Terms] OR "bacteria"[MeSH Terms]	2,768,968

Embase

Query	Results
(('bacteria'/exp OR bacteria) OR ('infectious agent'/exp OR 'infectious agent') OR microorganism* OR germ* OR microbe*) AND (('machine learning'/exp OR 'machine learning') OR ('computational intelligence'/exp OR 'computational intelligence') OR ('computer reasoning'/exp OR 'computer reasoning') OR ('computer vision system'/exp OR 'computer vision system') OR ('knowledge acquisition'/exp OR 'knowledge acquisition') OR 'knowledge representation' OR ('deep learning'/exp OR 'deep learning') OR ('neural network'/exp OR 'neural network') OR ('machine intelligence'/exp OR 'machine intelligence') OR ('artificial intelligence'/exp OR 'artificial intelligence')) AND (('infection control'/exp OR 'infection control') OR ('infection prevention'/exp OR 'infection prevention') OR 'contamination control' OR 'infection management' OR 'biosecurity measures' OR 'sanitation practices' OR 'hygiene control' OR 'pathogen control' OR 'epidemic control')	892
('infection control'/exp OR 'infection control') OR ('infection prevention'/exp OR 'infection prevention') OR 'contamination control' OR 'infection management' OR 'biosecurity measures' OR 'sanitation practices' OR 'hygiene control' OR 'pathogen control' OR 'epidemic control'	260087
'epidemic control'	1364
'pathogen control'	1025
'hygiene control'	274
'sanitation practices'	361
'biosecurity measures'	1138
'infection management'	2871
'contamination control'	796
'infection prevention'/exp OR 'infection prevention'	95281
'infection control'/exp OR 'infection control'	179305
('machine learning'/exp OR 'machine learning') OR ('computational intelligence'/exp OR 'computational intelligence') OR ('computer reasoning'/exp OR 'computer reasoning') OR ('computer vision system'/exp OR 'computer vision system') OR ('knowledge acquisition'/exp OR 'knowledge acquisition') OR 'knowledge representation' OR ('deep learning'/exp OR 'deep learning') OR ('neural network'/exp OR 'neural network') OR ('machine intelligence'/exp OR 'machine intelligence') OR ('artificial intelligence'/exp OR 'artificial intelligence')	1254162
'artificial intelligence'/exp OR 'artificial intelligence'	138746
'machine intelligence'/exp OR 'machine intelligence'	110677
'neural network'/exp OR 'neural network'	137675
'deep learning'/exp OR 'deep learning'	80915

'knowledge representation'	1345
'knowledge acquisition'/exp OR 'knowledge acquisition'	693514
'computer vision system'/exp OR 'computer vision system'	247
'computer reasoning'/exp OR 'computer reasoning'	66
'computational intelligence'/exp OR 'computational intelligence'	7275
'machine learning'/exp OR 'machine learning'	500159
('bacteria'/exp OR bacteria) OR ('infectious agent'/exp OR 'infectious agent') OR microorganism* OR germ* OR microbe*	7559328
microbe*	116804
germ*	5370892
microorganism*	318881
'infectious agent'/exp OR 'infectious agent'	41786
'bacteria'/exp OR bacteria	2305562

Web of science

Search Query	Results
((((ALL=(bacteria)) OR ALL=(pathogen*)) OR ALL=(microorganism*)) OR ALL=(germ*)) OR ALL=(microbe*)	9080736
(((((((((ALL=("machine learning")) OR ALL=("computational intelligence")) OR ALL=("computer reasoning")) OR ALL=("computer vision system")) OR ALL=("knowledge acquisition")) OR ALL=("knowledge representation")) OR ALL=("deep learning")) OR ALL=("neural network")) OR ALL=("machine intelligence")) OR ALL=("artificial intelligence")	1461552
((((((((ALL=("infection control")) OR ALL=("infection prevention")) OR ALL=("contamination control")) OR ALL=("infection management")) OR ALL=("biosecurity measures")) OR ALL=("sanitation practices")) OR ALL=("hygiene control")) OR ALL=("pathogen control")) OR ALL=("epidemic control")	59266
#1 AND #2 AND #3	77

S2: PRISMA-ScR template

Preferred Reporting Items for Systematic reviews and Meta-Analyses extension for Scoping Reviews (PRISMA-ScR) Checklist

SECTION	ITEM	PRISMA-ScR CHECKLIST ITEM	REPORTED ON PAGE #
**TITLE**			
Title	1	Identify the report as a scoping review.	1
**ABSTRACT**			
Structured summary	2	Provide a structured summary that includes (as applicable): background, objectives, eligibility criteria, sources of evidence, charting methods, results, and conclusions that relate to the review questions and objectives.	1
**INTRODUCTION**			
Rationale	3	Describe the rationale for the review in the context of what is already known. Explain why the review questions/objectives lend themselves to a scoping review approach.	1,2
Objectives	4	Provide an explicit statement of the questions and objectives being addressed with reference to their key elements (e.g., population or participants, concepts, and context) or other relevant key elements used to conceptualize the review questions and/or objectives.	2
**METHODS**			
Protocol and registration	5	Indicate whether a review protocol exists; state if and where it can be accessed (e.g., a Web address); and if available, provide registration information, including the registration number.	24
Eligibility criteria	6	Specify characteristics of the sources of evidence used as eligibility criteria (e.g., years considered, language, and publication status), and provide a rationale.	24
Information sources*	7	Describe all information sources in the search (e.g., databases with dates of coverage and contact with authors to identify additional sources), as well as the date the most recent search was executed.	24
Search	8	Present the full electronic search strategy for at least 1 database, including any limits used, such that it could be repeated.	24
Selection of sources of evidence†	9	State the process for selecting sources of evidence (i.e., screening and eligibility) included in the scoping review.	25, 26
Data charting process‡	10	Describe the methods of charting data from the included sources of evidence (e.g., calibrated forms or forms that have been tested by the team before their use, and whether data charting was done independently or in duplicate) and any processes for obtaining and confirming data from investigators.	26
Data items	11	List and define all variables for which data were sought and any assumptions and simplifications made.	26
Critical appraisal of individual sources of evidence§	12	If done, provide a rationale for conducting a critical appraisal of included sources of evidence; describe the methods used and how this information was used in any data synthesis (if appropriate).	25, 26
Synthesis of results	13	Describe the methods of handling and summarizing the data that were charted.	26
**RESULTS**			
Selection of sources of evidence	14	Give numbers of sources of evidence screened, assessed for eligibility, and included in the review, with reasons for exclusions at each stage, ideally using a flow diagram.	3

SECTION	ITEM	PRISMA-ScR CHECKLIST ITEM	REPORTED ON PAGE #
Characteristics of sources of evidence	15	For each source of evidence, present characteristics for which data were charted and provide the citations.	3-11
Critical appraisal within sources of evidence	16	If done, present data on critical appraisal of included sources of evidence (see item 12).	3-11
Results of individual sources of evidence	17	For each included source of evidence, present the relevant data that were charted that relate to the review questions and objectives.	3-11
Synthesis of results	18	Summarize and/or present the charting results as they relate to the review questions and objectives.	3-24
**DISCUSSION**			
Summary of evidence	19	Summarize the main results (including an overview of concepts, themes, and types of evidence available), link to the review questions and objectives, and consider the relevance to key groups.	23, 24
Limitations	20	Discuss the limitations of the scoping review process.	23, 24
Conclusions	21	Provide a general interpretation of the results with respect to the review questions and objectives, as well as potential implications and/or next steps.	23, 34
**FUNDING**			
Funding	22	Describe sources of funding for the included sources of evidence, as well as sources of funding for the scoping review. Describe the role of the funders of the scoping review.	27

JBI = Joanna Briggs Institute; PRISMA-ScR = Preferred Reporting Items for Systematic reviews and Meta-Analyses extension for Scoping Reviews.

* Where *sources of evidence* (see second footnote) are compiled from, such as bibliographic databases, social media platforms, and Web sites.

† A more inclusive/heterogeneous term used to account for the different types of evidence or data sources (e.g., quantitative and/or qualitative research, expert opinion, and policy documents) that may be eligible in a scoping review as opposed to only studies. This is not to be confused with *information sources* (see first footnote).

‡ The frameworks by Arksey and O'Malley (6) and Levac and colleagues (7) and the JBI guidance (4, 5) refer to the process of data extraction in a scoping review as data charting.

§ The process of systematically examining research evidence to assess its validity, results, and relevance before using it to inform a decision. This term is used for items 12 and 19 instead of "risk of bias" (which is more applicable to systematic reviews of interventions) to include and acknowledge the various sources of evidence that may be used in a scoping review (e.g., quantitative and/or qualitative research, expert opinion, and policy document).

From: Tricco AC, Lillie E, Zarin W, O'Brien KK, Colquhoun H, Levac D, et al. PRISMA Extension for Scoping Reviews (PRISMAScR): Checklist and Explanation. Ann Intern Med. 2018;169:467–473. doi: 10.7326/M18-0850

S3: Scopes of Aim definitions

The scopes included were Pathogen identification, Infection risk assessment, Therapeutic, Outbreak investigation, and Antimicrobial resistance stewardship.

Pathogen identification involves determining the specific microorganism causing an infection or disease. This process typically includes collecting samples from infected individuals, such as blood, tissue, or bodily fluids, and analyzing them using laboratory techniques like culture methods, polymerase chain reaction, and sequencing. Accurate identification is essential for understanding the nature of the pathogen, its transmission mechanisms, and for developing targeted treatments and control measures.

Infection risk assessment evaluates the likelihood of an infection spreading within a population. This involves assessing the risks and factors of an infection on a wide public health scale. Infection risk assessments are commonly used in healthcare settings, public health planning, and workplace safety to protect individuals and populations from infectious diseases.

The therapeutic scope focuses on developing and administering treatments to manage and cure infections. This includes researching and creating antibacterial medications, vaccines, and supportive care strategies. Effective therapeutic interventions are critical for reducing the severity and duration of illness, improving patient outcomes, and preventing complications.

Outbreak investigation involves systematically examining the origins and spread of an infectious disease within a community. Epidemiologists and public health professionals collect and analyze data to trace the source of the infection, identify affected individuals, and understand the transmission dynamics. This process includes field investigations, patient interviews, and mapping of infection patterns. The goal is to control the outbreak, implement measures to prevent future occurrences, and provide insights for better disease management.

Antimicrobial resistance and stewardship aims to optimize the use of antibiotics and other antimicrobial agents to combat resistance. This involves creating guidelines for appropriate prescribing, monitoring antibiotic use, and educating healthcare providers and the public about the dangers of overuse and misuse. Stewardship programs strive to preserve the effectiveness of existing treatments, reduce the incidence of resistant infections, and promote the development of new antimicrobial therapies.

S4: Scopes of Advantages definitions

Six themes of indication the advantages were generated and they were Enhanced Diagnostic Accuracy, Cost-Effectiveness and Efficiency, Improved Treatment Effectiveness, Predictive Modeling and Risk Assessment, Early Detection and Prevention, Data Utilization and Integration

Enhanced diagnostic accuracy refers to the ability of AI and machine learning models to improve the precision and reliability of medical diagnoses. Studies in this category demonstrate how these technologies can identify diseases and conditions more accurately than traditional methods. For instance, machine learning algorithms can analyze complex datasets, such as genomic data or medical imaging, to detect patterns and anomalies that may be missed by human clinicians. This leads to more accurate diagnoses, reducing the likelihood of misdiagnosis and ensuring that patients receive the appropriate treatment promptly. Enhanced diagnostic accuracy is crucial for improving patient outcomes and optimizing healthcare resources.

Cost-effectiveness and efficiency highlight the economic benefits and operational improvements brought about by AI and machine learning in healthcare. Studies under this theme show how these technologies can streamline clinical workflows, reduce the need for expensive diagnostic tests, and minimize hospital stays. By automating routine tasks and providing rapid, accurate analyses, AI systems can significantly cut down on healthcare costs. Additionally, efficient diagnostic processes mean that healthcare providers can serve more patients in less time, improving overall healthcare delivery. This theme underscores the potential of AI to make healthcare more affordable and accessible.

Improved treatment effectiveness focuses on how AI and machine learning can enhance the efficacy of medical treatments. This includes personalized medicine approaches where AI models predict the best treatment plans based on individual patient data, such as genetic information, medical history, and lifestyle factors. Studies in this category demonstrate how AI can optimize drug dosages, predict patient responses to treatments, and identify the most effective therapeutic strategies. By tailoring treatments to the specific needs of each patient, AI can improve recovery rates, reduce adverse effects, and enhance overall patient care.

Predictive modeling and risk assessment involve using AI and machine learning to forecast the likelihood of future health events and identify patients at high risk for certain conditions. Studies in this theme illustrate how predictive models can analyze vast amounts of data to predict disease outbreaks, patient deterioration, or the development of chronic conditions. These models enable healthcare providers to take proactive measures, such as early interventions or preventive care, to mitigate risks. Predictive modeling and risk assessment are essential for improving patient outcomes, reducing healthcare costs, and enhancing the overall quality of care.

Early detection and prevention focus on the ability of AI and machine learning technologies to identify diseases and health conditions at their earliest stages, often before symptoms become apparent. Studies in this category demonstrate how these technologies can analyze large datasets, including medical records, genetic information, and lifestyle data, to detect early signs of disease. By identifying at-risk individuals and potential health issues early, AI systems enable healthcare providers to implement preventive measures and early interventions. This can significantly reduce the progression of diseases, lower healthcare costs, and improve patient outcomes. Early detection and prevention are crucial for managing chronic diseases, reducing the incidence of acute health events, and promoting overall public health.

Data utilization and integration refer to the ability of AI and machine learning systems to effectively harness and combine diverse data sources for comprehensive analysis. Studies under this theme highlight how AI can integrate data from electronic health records, genomic databases, medical imaging, and other sources to provide a holistic view of patient health. This integrated approach allows for more informed decision-making, as clinicians can access a complete picture of a patient's health status. Data utilization and integration are critical for advancing precision medicine, improving diagnostic accuracy, and enhancing the overall efficiency of healthcare systems.

S5: Scopes of Limitations definitions

The six generated themes for the indication of limitations were Data Limitation, Lack of Real-World Validation, Limited Scope and Applicability, Complexity and Time-Consuming, Generalizability Issues, Technical and Computational Challenges

Data limitation refers to the constraints posed by insufficient, incomplete, or biased datasets used in AI and machine learning models. Studies under this theme highlight how the lack of comprehensive and high-quality data can hinder the development and accuracy of predictive models. Limited data can lead to overfitting, where the model performs well on training data but poorly on new, unseen data. This limitation can also result in models that fail to capture the full variability of real-world scenarios, reducing their effectiveness and reliability. Addressing data limitations is crucial for improving the robustness and generalizability of AI applications in healthcare.

Lack of real-world validation points to the gap between AI model performance in controlled research settings and their effectiveness in everyday clinical practice. Studies in this category emphasize that many AI models are validated using retrospective data or simulated environments, which may not accurately reflect real-world conditions. Without thorough validation in diverse, real-world settings, the reliability and applicability of these models remain uncertain. This limitation underscores the need for extensive clinical trials and real-world testing to ensure that AI systems can deliver consistent and accurate results in practical healthcare environments.

Limited scope and applicability refer to the narrow focus of some AI and machine learning models, which may be designed to address specific problems or datasets but lack broader utility. Studies under this theme illustrate how models tailored to particular conditions, populations, or data types may not be easily transferable to other contexts. This limitation can restrict the generalizability and scalability of AI solutions, making them less useful in varied clinical settings. Expanding the scope and applicability of AI models is essential for maximizing their impact across different healthcare domains.

Complexity and time-consuming processes highlight the challenges associated with developing, training, and deploying AI and machine learning models. Studies in this category point out that creating effective AI systems often requires significant computational resources, specialized expertise, and extensive time for data preprocessing, model training, and validation. These complexities can slow down the adoption of AI technologies in healthcare and make it difficult for smaller institutions with limited resources to implement them. Simplifying AI workflows and reducing the time and effort required for model development are critical for broader adoption and practical use.

Generalizability issues pertain to the difficulty of applying AI and machine learning models developed in one setting to different populations, environments, or conditions. Studies under this theme reveal that models trained on specific datasets may not perform well when applied to new, diverse datasets due to differences in patient demographics, clinical practices, or data collection methods. This limitation can lead to reduced accuracy and reliability of AI systems in real-world applications. Ensuring that models are trained on diverse and representative datasets is crucial for enhancing their generalizability and effectiveness across various healthcare settings.

Technical and computational challenges encompass the difficulties related to the implementation and operation of AI and machine learning systems. Studies in this category highlight issues such as the need for high computational power, the complexity of algorithm design, and the integration of AI systems with existing healthcare infrastructure. These challenges can pose significant barriers to the deployment

and scalability of AI technologies. Addressing technical and computational hurdles is essential for making AI solutions more accessible, efficient, and practical for widespread use in healthcare.
